# Phosphodiesterase 4 inhibition reduces lung fibrosis following targeted type II alveolar epithelial cell injury

**DOI:** 10.14814/phy2.13753

**Published:** 2018-06-27

**Authors:** Thomas H. Sisson, Paul J. Christensen, Yo Muraki, Anthony J. Dils, Lauren Chibucos, Natalya Subbotina, Kimio Tohyama, Jeffrey C. Horowitz, Takanori Matsuo, Marc Bailie, Sham Nikam, Masatoshi Hazama

**Affiliations:** ^1^ Pulmonary and Critical Care Division Department of Internal Medicine University of Michigan Medical Center Ann Arbor Michigan; ^2^ Division of Pulmonary & Critical Care Medicine Department of Internal Medicine William Beaumont Medical Center Troy Michigan; ^3^ Takeda Pharmaceutical Company Limited Fujisawa Japan; ^4^ In Vivo Facility Department of Pharmacology and Toxicology Michigan State University East Lansing Michigan

**Keywords:** cAMP, collagen, epithelium, fibroblast, pulmonary

## Abstract

Fibrosis of the lung constitutes a major clinical challenge and novel therapies are required to alleviate the associated morbidity and mortality. Investigating the antifibrotic efficacy of drugs that are already in clinical practice offers an efficient strategy to identify new therapies. The phosphodiesterase 4 (PDE4) inhibitors, approved for the treatment of chronic obstructive pulmonary disease, harbor therapeutic potential for pulmonary fibrosis by augmenting the activity of endogenous antifibrotic mediators that signal through cyclic AMP. In this study, we tested the efficacy of several PDE4 inhibitors including a novel compound (Compound 1) in a murine model of lung fibrosis that results from a targeted type II alveolar epithelial cell injury. We also compared the antifibrotic activity of PDE4 inhibition to the two therapies that are FDA‐approved for idiopathic pulmonary fibrosis (pirfenidone and nintedanib). We found that both preventative (day 0–21) and therapeutic (day 11–21) dosing regimens of the PDE4 inhibitors significantly ameliorated the weight loss and lung collagen accumulation that are the sequelae of targeted epithelial cell damage. In a therapeutic protocol, the reduction in lung fibrosis with PDE4 inhibitor administration was equivalent to pirfenidone and nintedanib. Treatment with this class of drugs also resulted in a decrease in plasma surfactant protein D concentration, a reduction in the plasma levels of several chemokines implicated in lung fibrosis, and an *in vitro* inhibition of fibroblast profibrotic gene expression. These results motivate further investigation of PDE4 inhibition as a treatment for patients with fibrotic lung disease.

## Introduction

Fibrosis of the lung interstitium occurs in response to multiple insults. Inhalation of organic and inorganic dusts and systemic exposure to specific medications including chemotherapy can all induce alveolar scarring. Lung fibrosis is also associated with a subset of rheumatologic diseases including systemic sclerosis and rheumatoid arthritis. In idiopathic pulmonary fibrosis (IPF), interstitial scarring occurs in the absence of an identifiable injury, although type II alveolar epithelial cell specific gene mutations are found in a subset of patients with both sporadic and familial forms of this disease (Wolters et al. 2014; Hambly et al. 2015). All these disorders that result in fibrosis of the lung interstitium are associated with significant morbidity and mortality. However, IPF arguably represents the greatest therapeutic challenge as this disease portends a median survival of only 3 years, and <20% of patients with this disease live beyond 8 years of their initial diagnosis (Wolters et al. 2014). The recent FDA approval of two new therapies for the treatment of IPF (pirfenidone and nintedanib) has resulted in guarded optimism, but there continues to be a desperate need for additional antifibrotic medications with increased efficacy and fewer side effects (Raghu et al. 2015; Spagnolo et al. 2015).

One approach to identify novel antifibrotic therapies is to investigate the efficacy of currently FDA‐approved agents that target specific profibrotic mechanisms. A significant advantage of this approach to repurpose existing compounds is the potential for rapid translation into patients because of the existing pharmacokinetic and pharmacodynamic data and safety studies in humans. As an example, phosphodiesterase 4 (PDE4) inhibitors including roflumilast, which is approved for the treatment of chronic obstructive pulmonary disease (COPD), hold promise as antifibrotic agents. This class of drugs blocks the degradation of cAMP and thereby augments the activity of antifibrotic mediators that signal through G‐protein‐coupled receptors including prostaglandin E_2_ (PGE_2_), prostacyclin, and adenosine. PGE_2_ exhibits a multitude of antifibrotic effects including inhibiting fibroblast activation, increasing fibroblast susceptibility to apoptosis, and maintaining alveolar epithelial cell integrity (Thomas et al. 2007; Huang et al. 2009; Maher et al. 2010; Penke et al. 2014). The antifibrotic potential of PDE4 inhibitors is supported by several *in vitro* studies. Specifically, the PDE4 inhibitor piclamilast was found to blunt transforming growth factor‐beta (TGF‐*β*)‐mediated fibroblast to myofibroblast differentiation, an effect that was accentuated by the addition of exogenous PGE_2_ (Dunkern et al. 2007). In a separate *in vitro* study, roflumilast inhibited both TGF‐*β* stimulated fibroblast contraction of three‐dimensional collagen gels and fibroblast chemotaxis toward fibronectin (Togo et al. 2009). Importantly, roflumilast also increased PGE_2_ release by fibroblasts. PDE4 inhibitors have also been found to mitigate bleomycin‐induced fibrosis *in vivo*. Specifically, the initiation of roflumilast treatment on day 0 and day 7 following bleomycin injury reduced day 14 and day 21 lung fibrosis, respectively (Cortijo et al. 2009). In a separate study, roflumilast also reversed the metabolic alterations of bleomycin injury when administered in a preventative regimen (Milara et al. 2015).

The aforementioned studies supporting the antifibrotic potential of PDE4 inhibitors led us to further investigate the efficacy of this class of medications in a murine model of lung fibrosis that results from a targeted insult to the type II alveolar epithelium (Sisson et al. 2010). We hypothesized that both preventative and therapeutic PDE4 inhibitor administration protocols would mitigate pulmonary fibrosis in this model. Type II alveolar epithelial cell injury‐induced fibrosis differs from bleomycin in that the inflammatory response is modest (marked by the selective accumulation of Ly‐6c high monocytes and exudate macrophages) and the resultant fibrosis is more diffuse (Sisson et al. 2010; Osterholzer et al. 2012, 2013). PDE4 inhibitors including piclamilast, roflumilast, and a novel agent (Compound 1) were tested and the efficacy of PDE4 inhibition was compared to pirfenidone and nintedanib. The effects of PDE4 inhibition on lung chemokine levels, whole‐lung fibrogenic gene expression, and plasma surfactant protein D (SP‐D) levels (as an indicator of epithelial cell integrity) were also measured (Hambly et al. 2015).

We found that both prophylactic and therapeutic administration of PDE4 inhibitors including the novel agent Compound 1 significantly reduced lung fibrosis following type II AEC injury with an efficacy comparable to pirfenidone and nintedanib. The protection against lung fibrosis was associated with diminished weight loss and a significant decline in plasma SP‐D levels. These findings highlight the antifibrotic potential of PDE4 inhibition, and with roflumilast already FDA approved for the treatment of COPD and additional novel agents on the horizon, our data motivate the clinical investigation of this class of drugs in patients with IPF and other fibrotic lung diseases.

## Methods

### Animals

All animal experiments were performed in accordance with institutional guidelines set forth by the University Committee on the Use and Care of Animals at the University of Michigan. Transgenic mice expressing the diphtheria toxin receptor (DTR) under the control of the murine SPC promoter were generated in our laboratory on a C57BL/6 background (designated DTR+ mice) (Sisson et al. 2010). The presence of the DTR was detected using PCR for the SPC‐DTR transgenic construct as previously described (Sisson et al. 2010). Control mice included littermates of SPC‐DTR transgenic mice that were PCR‐negative for the transgenic construct and C57BL/6 mice purchased from Jackson Laboratories (Bar Harbor, ME). These control animals are designated DTR‐ and prior studies revealed that neither of these strains of mice develop significant fibrosis in response to diphtheria toxin (DT) administration.

### Drug concentration and cAMP levels

Roflumilast (5 mg/kg) and Compound 1 (10 mg/kg) were administered to C57BL/6N mice (Charles River Laboratories Japan, Inc., Japan) which were then sacrificed at 0.5, 1, 2, 4, 8, and 24 h after the dosing. Blood samples were collected and centrifuged to obtain plasma specimens. Plasma concentrations of roflumilast, the active metabolite of roflumilast N‐oxide and Compound 1 were measured using liquid chromatography/tandem mass spectrometry. Bronchoalveolar Lavage Fluid (BALF) was collected by instilling three aliquots of 0.5 mL 0.9% saline into the lungs. BALF was centrifuged to remove cells, the supernatant was deproteinized with trichloroacetic acid, and washed by diethylether. The aqueous layer was freeze‐dried and dissolved by lysis buffer enclosed in cAMP XP Assay kit (Cell Signaling Technology Inc., Danvers, MA). cAMP content was measured in accordance with the manufacturer's instruction, and, total PDE4 inhibitory activities (tPDE4i) were calculated by following equation:

$$\text{tPDE4i}=\sum \frac{\left(C_{n}\;\text{or}\;{\text{AUC}}_{n}\right)\times {\text{fu}}_{n}}{{\text{IC50}}_{n}}$$

where *C*_*n*_ and AUCn represent the plasma concentration and the AUC of active pharmacological ingredient (API), respectively, fu is the unbound fraction in plasma, IC50 is the concentration resulting in 50% inhibition in an *in vitro* assay and n represents the number of APIs (Hermann et al. 2007). Of note, roflumilast has two APIs and Compound 1 has one API.

### Preparation of test compounds

All drugs were suspended in 0.5% methylcellulose and administered by oral gavage. Roflumilast (1.0 and 5.0 mg/kg), piclamilast (30 mg/kg) and Compound 1 (N‐[Amino(dimethylamino)methylidene]‐4‐[(3aS,9bR)‐8‐ethoxy‐7‐methoxy‐1,3,3a,9b‐tetrahydrofuro[3,4‐c]isoquinolin‐5‐yl]benzamide at 1.0, 5.0, and 10.0 mg/kg) were given once daily by gavage. Pirfenidone was dosed at 100 mg/kg three times daily (Liu et al. 2017) and nintedanib was dosed at 100 mg/kg twice daily (Wollin et al. 2014) by gavage.

### Diphtheria toxin administration and experimental design

Six‐ to ten‐week‐old mice were intraperitoneally injected with DT (Sigma Chemical, St. Louis, MO) once daily for 14 days at a dose of 12.5 *μ*g/kg in 100 *μ*L of PBS. Control groups received intraperitoneal PBS alone. Prophylactic dosing of the experimental treatments was initiated on day 1 and continued daily at the indicated doses throughout the 21‐day study protocol. Therapeutic dosing was initiated on day 11 and continued daily at the indicated doses for 10 days. Mice were weighed daily and, on day 21 of the study protocol (7 days after the last dose of DT), blood and lungs were harvested 1 h after the last drug administration. Lungs were either sectioned for histology or homogenized for hydroxyproline analysis and whole lung gene expression. Plasma was separated from whole blood and analyzed for chemokine and SP‐D levels.

### Hydroxyproline assay

Hydroxyproline content of both lungs was measured as previously reported (Sisson et al. 2010).

### Lung histology

The left lung was inflation‐fixed at 25 cm H_2_O pressure with 10% neutral‐buffered formalin, removed en bloc, further fixed in 10% neutral‐buffered formalin overnight, and then paraffin embedded. Five micron sections were stained using picrosirius red.

### Plasma SP‐D levels

Plasma SP‐D levels were measured using SP‐D ELISA Kit (Yamasa Corp., Japan). Samples were diluted using the provided buffer and measurements were made in accordance with the manufacturer's protocol.

### Plasma Chemokine analysis

Plasma chemokine concentrations were measured using the Milliplex MAP mouse cytokine/chemokine magnetic bead panel kit (Merck Millipore Corp., Germany) according to the manufacturer's instructions.

### *In vitro* fibroblast studies

For cAMP assay, WI‐38 human lung fibroblasts were suspended in E‐MEM (Thermo Fisher Scientific Inc., MA) containing 10% heat‐inactivated fetal bovine serum (FBS) and seeded on 24‐well plates at 50,000 cells/500 *μ*L/well. Twenty‐four hours after seeding, cells were treated with Compound 1 (1 × 10^‐10^ mol/L to 1 × 10^‐5^ mol/L) dissolved in HBSS (Thermo Fisher Scientific Inc.) containing 0.1% bovine serum albumin (BSA) and 5 mmol/L HEPES. After 30 min, cells were stimulated by 1 *μ*mol/L forskolin for 30 min and then lysed. cAMP was measured in accordance with the cAMP XP Assay kit protocol (Cell Signaling Technology Inc.).

To analyze fibroblast gene expression, WI‐38 human lung fibroblasts were suspended in E‐MEM (Thermo Fisher Scientific) containing 10% heat‐inactivated FBS and seeded on 24 well plates at 50,000 cells/500 *μ*L/well. After seeding, cells were cultured in E‐MEM containing 0.5% FBS for 24 h. The cultures were then treated with Compound 1 (1 × 10^‐10^ mol/L to 1 × 10^‐5^ mol/L) for 1 h followed by TGF‐*β* (3 ng/mL) and forskolin (1 *μ*mol/L) for 24 h. Total RNA was extracted from cell lysate using RNeasy 96 Kit (QIAGEN, Germany). cDNA was amplified using High‐Capacity cDNA Reverse Transcription Kit (Thermo Fisher Scientific), and target gene mRNA was measured using TaqMan PCR (7900HT Thermo Fisher Scientific). The target gene expression level was normalized by mRNA expression of Glyceraldehyde 3‐phosphate dehydrogenase (GAPDH). TaqMan gene expression assays (Thermo Fisher Scientific) were used to measure gene expression. Assay ID of human GAPDH, type‐1 collagen, fibronectin, connective tissue growth factor, and plasminogen activator inhibitor‐1 were Hs02758991g1, Hs00164004m1, Hs00365052m1, Hs01026927g1, and Hs00167155m1, respectively.

### *In vitro* A549 cell studies

For cAMP assays, A549 human lung epithelial cells were suspended in Ham's F‐12K (Thermo Fisher Scientific Inc.) containing 10% heat‐inactivated FBS and seeded on 24 well plates as 50,000 cells/500 *μ*L/well for 24 h. Cells were treated with Compound 1 (1 × 10^‐10^ mol/L to 1 × 10^‐5^ mol/L) dissolved in HBSS (Thermo Fisher Scientific Inc.) containing 0.1% bovine serum albumin (BSA) and 5 mmol/L HEPES for 30 min and stimulated with 10 *μ*mol/L forskolin and 10 *μ*mol/L prostaglandin E2 for 30 min. Cells were lysed and cAMP content in cell lysates and supernatants were measured using cAMP XP Assay kit (Cell signaling technology, MA).

### Quantitative RT‐PCR on lung homogenates

RNA was isolated from the left lung using the RNeasy Plus Mini Kit (Qiagen) and first‐strand cDNA was synthesized using SuperScriptIII (Invitrogen, Carlsbad, CA). Specific qPCRs with SYBR Green‐based detection using an MX 3000P system (Stratagene, La Jolla, CA) were conducted for forty cycles (94°C for 15 sec followed by 60°C for 30 sec and 72°C for 30 sec) using each cDNA template. The mRNA levels were normalized with the expression of GAPDH using the following formula: %GAPDH expression = 100/2‐ΔΔCT. Primer pairs for murine collagen1a1, tumor necrosis factor *α*, fibronectin, connective tissue growth factor, and plasminogen activator inhibitor 1 were purchased from Applied Biosystems.

### Statistical analysis

All data were expressed as mean ± SEM. Data were evaluated by unpaired Student's *t*‐test (for comparison between two samples), a two‐tailed William's test, one‐way ANOVA with a Tukey multiple comparisons test, or a two‐way ANOVA with a Tukey multiple comparisons test. A statistically significant difference was accepted at *P* < 0.05.

## Results

### Pharmacokinetics of the phosphodiesterase 4 inhibitors

**Figure 1:**
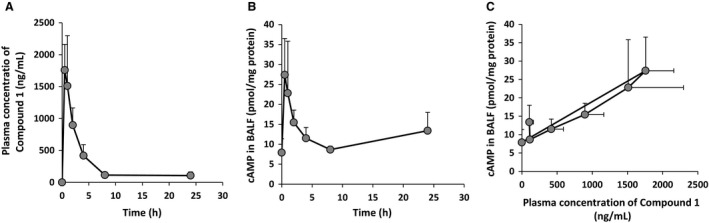
The pharmacokinetics of Compound 1 administration. 10.0 mg/kg of Compound 1 was administered by oral gavage at time 0. Plasma and BALF were collected simultaneously from separate cohorts of mice at 0.5, 1, 2, 4, 8, and 24 h after the dosing. The plasma concentration of drug over time (1A) and the BALF cAMP levels over time (1B) are reported. A linear relationship between plasma drug level and BALF cAMP concentration was observed (1C). Data are reported as mean ± SD (*n* = 4/group).

Prior to testing the efficacy of PDE4 inhibitors in our murine model of lung fibrosis, we assessed the pharmacokinetics of Compound 1 with our planned dosing regimen in uninjured wild‐type mice. Compound 1 is a novel PDE4 inhibitor with a different structural scaffold than Roflumilast and Piclamilast, and in contrast to Piclamilast and Roflumilast which both inhibit CYP3A4, Compound 1 has no CYP inhibitory activity. For Compound 1, a single dose of (10.0 mg/kg) was administered by oral gavage at time 0. Plasma was then collected at 0.5, 1.0, 2.0, 4.0, 8.0, and 24.0 h. Bronchoalveolar lavage fluid (BALF) was collected simultaneously and analyzed for cAMP levels. As demonstrated in Figure 1A, the 10.0 mg/kg dose of Compound 1 resulted in a peak plasma concentration at 0.5 h of 1585.4 tPDE4i. The plasma level then decreased over the next 8 h to a level of 96.1, and this low PDE4i of drug persisted at 24 h. The BALF concentration of cAMP correlated with the drug level of the PDE4 inhibitor with a peak level of 27.4 pmol/mg protein occurring at the 0.5 h time point (up from a basal concentration of 7.9 pmol/mg protein) (Fig. 1B). By 8 h, the BALF concentration of cAMP had returned to basal levels. Figure 1C demonstrates the linear increase in the BALF concentration of cAMP in direct relation to the increasing drug level of Compound 1 suggesting that BALF cAMP concentration can be used as a marker of drug effect in the target tissue.

Similar pharmokinetics were observed following a single administration of roflumilast at 5.0 mg/kg (data not shown). The peak concentration was measured at 0.5 h and returned to baseline at 8 h. As was the case with Compound 1, the BALF concentration of cAMP correlated with the drug level of the PDE4 inhibitor with a peak level of 33.3 pmol/mg protein occurring at the 0.5 h time point (up from a basal concentration of 10.9 pmol/mg protein) and returning near baseline by 8 h (data not shown).

### Prophylactic administration of piclamilast and roflumilast in the type II AEC injury model of lung fibrosis

**Figure 2:**
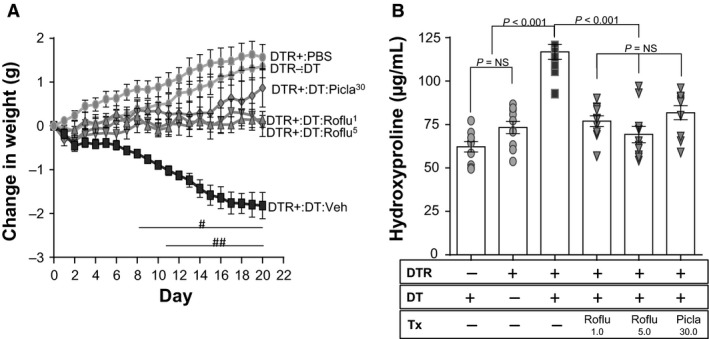
Prophylactic administration of roflumilast and piclamilast to targeted type II AEC‐injured mice reduces weight loss and ameliorates pulmonary fibrosis. DTR‐expressing mice (DTR+) were administered daily I.P. PBS or DT from day 0 through Day 14. Subsets of the DTR+:DT‐injured animals were treated by oral gavage once daily beginning on day 0 with vehicle, roflumilast (1.0 mg/kg or 5.0 mg/kg), or Piclamilast at 30 mg/kg. Mice were weighed daily (A) and on day 21, lungs were harvested and analyzed for total collagen content using an assay for hydroxyproline (B). Data are reported as mean ± SEM (*n* = 10 per group). Weight curves are analyzed with a two‐way ANOVA + Tukey multiple comparison tests (^#^ *P* ≤ 0.05 for DTR+:DT Piclamilast‐treatment versus DTR+:DT vehicle treatment. ^##^ *P* ≤ 0.05 DTR+:DT piclamilast and roflumilast (1.0 mg/kg or 5.0 mg/kg) treatment versus DTR+:DT vehicle treatment), and hydroxyproline is analyzed with a one‐way ANOVA + Tukey multiple comparison tests.

To determine if PDE4 inhibition would mitigate pulmonary fibrosis that results from targeted type II AEC injury, we treated DTR+ mice with daily doses of intraperitoneal DT for 14 days. In previous studies, we demonstrated that this dosing regimen induced a progressive weight loss and lung fibrosis that was readily detected by day 21, 1 week after the last DT injection (Sisson et al. 2010). To assess the efficacy of PDE4 inhibition in this model, we initially administered a prophylactic dosing regimen of piclamilast (30 mg/kg daily) or roflumilast (1.0 and 5.0 mg/kg) via oral gavage beginning on day 0 and continuing through day 21 of the model. By treating throughout the 21 day experimental time course, we sought to optimize our ability to detect a significant effect of PDE4 inhibition in our fibrosis model. We found that treatment of DTR+ mice with either piclamilast or roflumilast blunted the decline in body weight due to DT administration (Fig. 2A). By day 11, the protection against weight loss afforded by these two drugs was statistically significantly different when compared with the vehicle‐treated DTR+‐DT injured mice. Treatment with either drug (and for roflumilast, both doses) resulted in a significant reduction in lung collagen content as measured by hydroxyproline compared to DT‐injured DTR+ mice that were treated with vehicle (Fig. 2B). The resulting level of lung hydroxyproline following PDE4 inhibition was similar to the uninjured control groups (i.e., DTR‐ mice treated with DT and DTR+ mice treated with PBS).

### Prophylactic administration of Compound 1 and piclamilast in the type II AEC injury model of lung fibrosis

**Figure 3:**
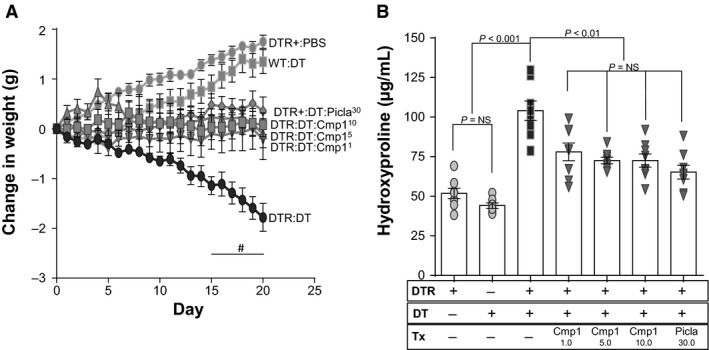
Prophylactic administration of Compound 1 to targeted type II AEC‐injured mice reduces weight loss and ameliorates pulmonary fibrosis. DTR‐expressing mice (DTR+) were administered daily I.P. PBS or DT from day 0 through Day 14. Subsets of the DTR+:DT‐treated animals were treated by oral gavage once daily beginning on day 0 with vehicle, Compound 1 (1.0 mg/kg, 5.0 mg/kg, or 10.0 mg/kg) or Piclamilast at 30 mg/kg. Mice were weighed daily (A) and on day 21, lungs were harvested and analyzed for total collagen content using an assay for hydroxyproline (B). Data are reported as mean ± SEM (*n* = 8 per group). Weight curves are analyzed with a two‐way ANOVA + Tukey multiple comparison tests (^#^ *P* ≤ 0.05 for DTR+:DT piclamilast and Compound 1‐treatments (all doses) versus DTR+:DT vehicle treatment), and hydroxyproline is analyzed with a one‐way ANOVA + Tukey multiple comparison tests.

After confirming the efficacy of prophylactic PDE4 inhibition with piclamilast and roflumilast in reducing fibrosis following targeted type II AEC injury, we next assessed whether the novel PDE4 inhibitor Compound 1 would also protect against fibrosis in our murine model. Compound 1 was administered daily at doses of 1.0 mg/kg, 5.0 mg/kg, and 10.0 mg/kg from day 0 through day 21. The efficacy of this molecule was compared to daily treatment with piclamilast (30 mg/kg). We found that all doses of Compound 1 lessened the DT‐injury‐induced weight loss in DTR+ mice, and the protection afforded by this agent (at each dose) was comparable to piclamilast (Fig. 3A). By day 15, the weight losses in the groups of DTR+‐DT injured mice receiving the three doses of Compound 1 were significantly less than in the vehicle‐treated DTR+‐DT injured group. All doses of Compound 1 also significantly reduced the day 21 lung collagen content when compared with DT‐injured DTR+ mice that were treated with vehicle (Fig. 3B). Also, the levels of lung hydroxyproline in the three dose groups of Compound 1 were not statistically different from the group that was treated with 30 mg/kg of piclamilast.

### Therapeutic administration of Compound 1 and roflumilast in the type II AEC injury model of lung fibrosis

**Figure 4:**
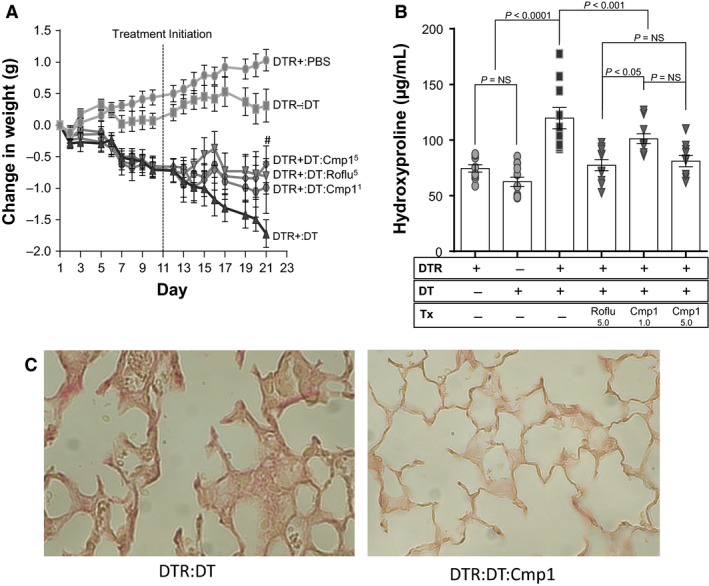
Therapeutic administration of Compound 1 and roflumilast to targeted type II AEC‐injured mice reduces weight loss and ameliorates pulmonary fibrosis. DTR‐expressing mice (DTR+) were administered daily I.P. PBS or DT from day 0 through Day 14. Subsets of the DTR+:DT‐treated animals were treated by oral gavage once daily beginning on day 11 with vehicle, roflumilast (5.0 mg/kg), or Compound 1 (1.0 mg/kg or 5.0 mg/kg). Mice were weighed daily (A) and on day 21, lungs were harvested and analyzed for total collagen content using an assay for hydroxyproline (B) and by histopathology (C) with representative sections stained with picrosirius red (400× magnification). Data are reported as mean ± SEM (*n* = 10–12 per group). Weight curves are analyzed with a two‐way ANOVA + Tukey multiple comparison tests (^#^ *P* ≤ 0.05 for DTR+:DT roflumilast and Compound 1 (5.0 mg/kg) treatments versus DTR+:DT vehicle treatment), and hydroxyproline is analyzed with a one‐way ANOVA + Tukey multiple comparison tests.

We next tested whether PDE4 inhibition would also diminish lung fibrosis when delivered in a therapeutic protocol. For these experiments, DTR+ mice were administered daily doses of DT from day 1 through day 14. From day 11 through day 21, subsets of mice were treated with Compound 1 at doses of 1.0 mg/kg or 5.0 mg/kg, roflumilast at 5.0 mg/kg, or vehicle. The time point of treatment initiation was chosen based on our prior studies which demonstrated that the onset of fibrosis was detectable around day 11 and that 10 days of treatment was sufficient to detect a therapeutic effect of a previously studied antifibrotic drug (Sisson et al. 2015). Weight loss was tracked throughout the study, and the severity of lung fibrosis was assessed on day 21 by lung hydroxyproline concentration and picrosirius red‐stained histologic sections. At the time of treatment initiation on day 11, the mean body weight was equivalent in all DT‐treated DTR+ cohorts. Treatment with the PDE4 inhibitors limited the further decline in body weight that was observed in the vehicle‐treated group from day 11 through day 21 (Fig. 4A, *P* ≤ 0.05 on day 21 for Compound 1 (5.0 mg/kg) and Roflumilast (5.0 mg/kg)‐treated mice compared to the vehicle‐treated group). In the 5.0 mg/kg doses of Compound 1 and roflumilast groups, the PDE4 inhibitor‐mediated protection against weight loss was associated with a significant reduction in lung collagen content (Fig. 4B). Of note, the 1.0 mg/kg therapeutic treatment dose of Compound 1 did not significantly reduce lung collagen content compared to the group that received vehicle, suggesting that this dose is below the efficacy threshold. The therapeutic administration of Compound 1 at 5.0 mg/kg also substantially reduced picrosirius red staining (specific for collagen) and alveolar wall thickening characteristic of the histologic abnormalities observed in DT‐injured DTR+ mice on day 21 (Fig. 4C).

### Compound 1 increases cAMP levels in A549 cells and reduces plasma surfactant protein D levels

**Figure 5:**
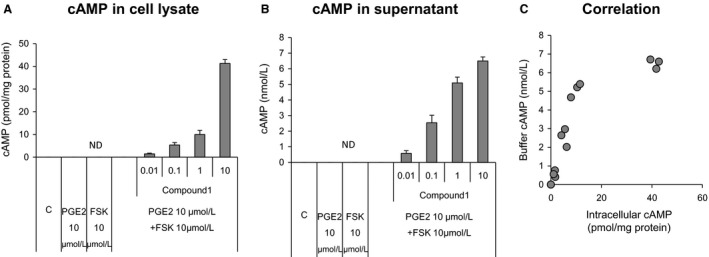
Compound 1 treatment of A549 human lung epithelial cell cultures increases intracellular and extracellular cAMP concentrations. A549 human lung epithelial cells were seeded in a 24 well plate for 24 h. The cultures were treated with increasing concentrations of Compound 1 (1 × 10^−10^ mol/L to 1 × 10^−5^ mol/L) for 30 min and stimulated with 10 *μ*mol/L forskolin and 10 *μ*mol/L prostaglandin E2 for 30 min. cAMP concentrations were measured in cell lysates (A) and culture supernatants (B). The correlation between intracellular cAMP and cAMP in the culture supernatants is shown in (C). Data are reported as mean + SD (*n* = 3 per group).

**Figure 6:**
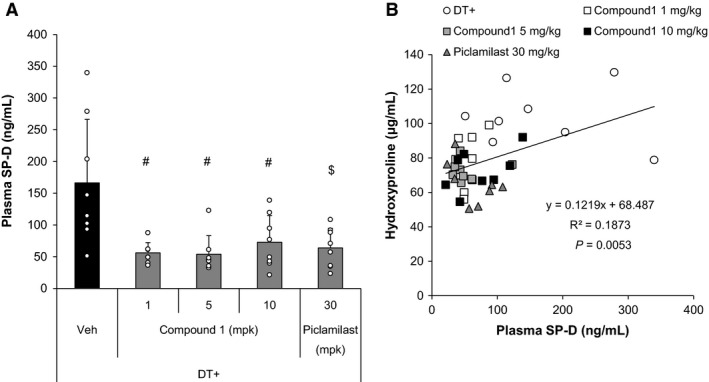
Prophylactic treatment of targeted type II AEC‐injured mice with Compound 1 reduces plasma SP‐D levels. DTR‐expressing mice (DTR+) were administered daily I.P. PBS or DT from day 0 through Day 14. Subsets of the DTR+:DT‐treated animals were treated by oral gavage once daily beginning on day 0 with vehicle, Compound 1 (1.0 mg/kg, 5.0 mg/kg, or 10.0 mg/kg) or Piclamilast at 30 mg/kg. Plasma was collected on day 21 and analyzed for SP‐D concentration (A). The plasma SP‐D concentration was then correlated with lung collagen content (B). Data are reported as mean + SEM with individual data points shown (*n* = 8 per group). ^#^ *P* ≤ 0.05 versus Vehicle group by two‐tailed Williams’ test. ^$^ *P* ≤ 0.05 versus Vehicle group by Student's *t*‐test.

After establishing both prophylactic and therapeutic efficacy of PDE4 inhibition in mitigating type II AEC‐injury‐induced lung fibrosis, we performed studies to gain insight into the mechanisms by which this class of drugs exerts its antifibrotic effect. We first examined whether alveolar epithelial cells were responsive to PDE4 inhibition by measuring the level of cAMP following Compound 1 treatment. For these studies, A549 cells, a malignant human alveolar epithelial cell line, were treated with increasing doses of Compound 1 (in the presence of 10 *μ*mol/L PGE2 and 10 *μ*mol/L forskolin to stimulate cAMP production). As demonstrated in Figure 5A and B, Compound 1 induced a dose‐dependent increase in cAMP concentrations in both cell lysates and conditioned media. At lower concentrations of intracellular cAMP (below 20 pmol/mg protein), a linear relationship existed between intracellular and extracellular levels of this mediator (Fig. 5C). After confirming activity in A549 cells, we examined whether Compound 1 (1.0 mg/kg, 5.0 mg/kg and 10.0 mg/kg) or piclamilast (30 mg/kg) modulated alveolar epithelial cell integrity following targeted type II AEC injury by measuring day 21 plasma surfactant protein D (SP‐D) levels. Using the prophylactic treatment protocol, we found that all three doses of Compound 1 and the 30 mg/kg dose of piclamilast significantly reduced plasma SP‐D compared to DT‐injured DTR+ mice treated with vehicle (Fig. 6A). Of note, there was a weak but statistically significant correlation between the plasma SP‐D level at day 21 and lung collagen content in individual mice (*r* ^2^ = 18.7%, *P* = 0.0053) (Fig. 6B).

### Compound 1 treatment decreases the plasma level of select chemokines

**Figure 7:**
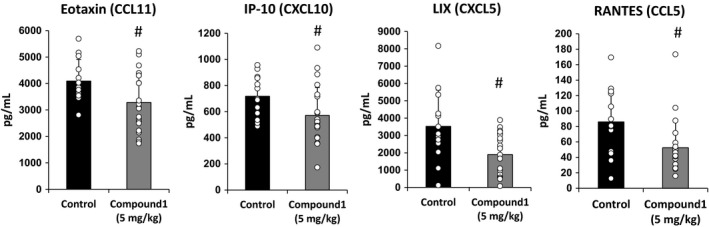
Therapeutic treatment of targeted type II AEC‐injured mice with Compound 1 reduces plasma cytokine levels. DTR‐expressing mice (DTR+) were administered daily I.P. PBS or DT from day 0 through Day 14. Subsets of the DTR+:DT‐treated animals were treated by oral gavage once daily beginning on day 0 with vehicle or Compound 1 at 5.0 mg/kg. Plasma was collected on day 21 and analyzed for CCL11/eotaxin, CXCL10/IP‐10, CXCL5/LIX, and CCL5/Rantes. Data are reported as mean ± SD with individual data points shown (*n* = 13 for control, 22 for Compound 1 per group). ^#^ *P* ≤ 0.05 versus Control group by Student's *t*‐test.

As another potential mechanism by which PDE4 inhibition reduces lung fibrosis in the setting of targeted type II AEC injury, we determined whether Compound 1 altered plasma levels of a select subset of chemokines. For these studies DT‐injected DTR mice were treated with a therapeutic administration of Compound 1 at 5.0 mg/kg. Plasma was collected on day 21, and levels of eotaxin (CCL11), IP‐10 (CXCL10), LIX (CXCL5) and Rantes (CCL5) were measured by bead‐based multianalyte profiling. We found that PDE4 inhibition with Compound 1 reduced the concentration of all of these mediators with the greatest effect on CXCL5 and CCL5 (Fig. 7).

### Compound 1 treatment decreases fibrogenic gene expression in fibroblasts *in vitro* and blunts TNF*α* expression *in vivo*

**Figure 8:**
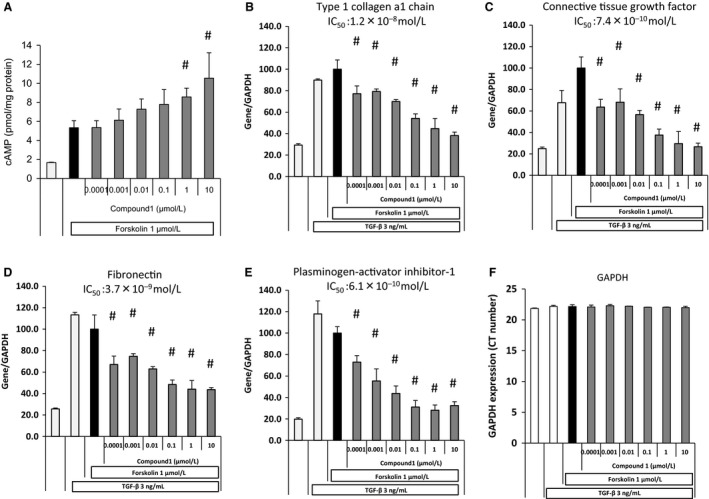
Compound 1 treatment of human lung fibroblasts cell cultures increases intracellular cAMP and decreases TGF‐*β*‐mediated induction of fibrotic gene expression. WI‐38 human lung fibroblasts were seeded on 24‐well plates and treated with increasing concentrations of Compound 1 (1 × 10^−10^ mol/L to 1 × 10^−5^ mol/L). After 30 min, cells were stimulated by 1 *μ*mol/L forskolin for 30 min and cell lysates were analyzed for cAMP concentration (A). To analyze fibroblast gene expression, WI‐38 human lung fibroblasts were cultured in media containing 0.5% FBS for 24 h and treated with increasing concentrations of Compound 1 (1 × 10^−10^ mol/L to 1 × 10^−5^ mol/L) for 1 h followed by TGF‐*β* (3 ng/mL) and forskolin (1 *μ*mol/L) for 24 h. Total RNA was extracted from cell lysates, cDNA was amplified, and target gene mRNA was measured. The target gene expression levels of Col1a1 (Type‐1 collagen), Fn (Fibronectin), CTGF (connective tissue growth factor), and PAI‐1 (plasminogen activator inhibitor‐1) was normalized to Glyceraldehyde 3‐phosphate dehydrogenase (GAPDH). Data are reported as mean ± SD with individual data points shown (*n* = 3 per group). ^#^ *P* ≤ 0.05 versus forskolin group by two‐tailed Williams’ test.

Fibroblasts and myofibroblasts are considered the primary effector cells in tissue fibrosis. These cells are the primary source of key extracellular matrix constituents including the fibrillar collagens (including collagen 1) and fibronectin (Fn). These cells also secrete other profibrotic molecules including plasminogen activator inhibitor 1 (PAI‐1) and connective tissue growth factor (CTGF). Prior studies have demonstrated that PDE4 inhibitors impede certain fibroblast functions including three‐dimentional gel contraction, migration, and myofibroblast differentiation (Dunkern et al. 2007; Togo et al. 2009). To assess whether PDE4 inhibition also influenced fibroblast expression of profibrotic mediators, WI‐38 human lung fibroblasts were treated with increasing doses of Compound 1 in the presence of forskolin (1.0 *μ*mol/L) and transforming growth factor‐*β* (TGF‐*β*, 3.0 ng/mL). We then measured the expression levels of Fn, collagen 1*α*1, PAI‐1, and CTGF. Exposure of WI‐38 cells to Compound 1 in the presence of forskolin increased the level of intracellular cAMP in a dose‐dependent manner (Fig. 8). This increase in intracellular cAMP reduced the TGF‐*β* stimulated expression of Fn, collagen 1*α*1 chain, PAI‐1, and CTGF in a dose‐dependent manner (Fig. 8).

**Figure 9:**
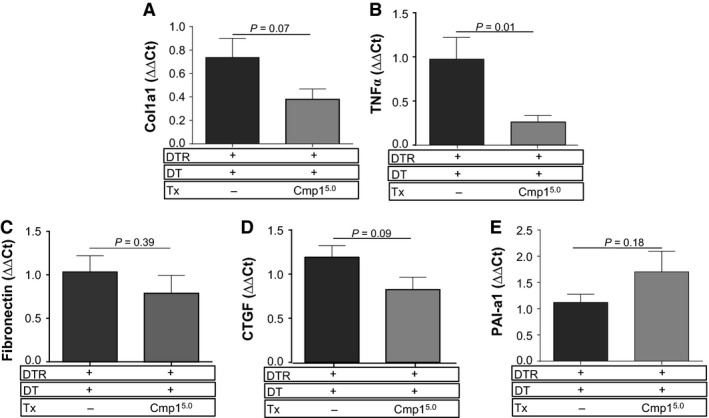
Therapeutic administration of Compound 1 to targeted type II AEC‐injured mice reduces the expression of TNF *α* within the lung. DTR‐expressing mice (DTR+) were administered daily I.P. PBS or DT from day 0 through Day 14. Subsets of the DTR+:DT‐treated animals were treated by oral gavage once daily beginning on day 11 with vehicle or Compound 1 at 5.0 mg/kg. On day 21, the left lung was harvested and homogenized, and total RNA was extracted. First‐strand cDNA was synthesized and mRNA levels for Col1a1, Fibronectin, CTGF TNF *α* and PAI‐1 (plasminogen activator inhibitor‐1) were assessed using SYBR Green‐based detection. The expression levels were normalized to GAPDH using the following formula: %GAPDH expression = 100/2‐ΔΔCT. Data are presented as an average ± SEM (*n* = 9 per DTR+:DT vehicle‐ and drug‐treated groups).

To determine if treatment with Compound 1 also inhibited profibrotic gene expression *in vivo*, DT‐injured DTR+ mice were treated with 5.0 mg/kg of Compound 1 from day 11 through day 21. On day 21, RNA was purified from lung tissue, and message levels for murine TNF*α*, collagen 1*α*1, PAI‐1, CTGF, and Fn were assessed by quantitative RT‐PCR. We found that only TNF*α* expression was significantly reduced at the day 21 time point (Fig. 9B). The mean expression of collagen 1*α*1, CTGF, and Fn were also decreased, but the reduction did not achieve statistical significance from the vehicle‐treated DT‐injured DTR+ group (Fig. 9A,C and D).

### The therapeutic efficacy of PDE4 inhibition versus pirfenidone and nintedanib

**Figure 10:**
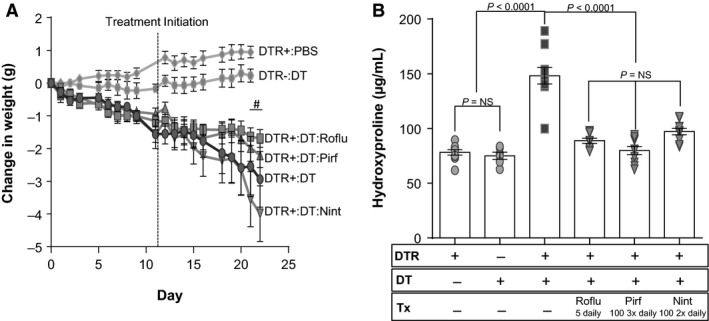
Roflumilast is equivalent to pirfenidone and nintedanib in ameliorating fibrosis following targeted type II AEC injury. DTR‐expressing mice (DTR+) were administered daily I.P. PBS or DT from day 0 through Day 14. Subsets of the DTR+:DT‐treated animals were treated by oral gavage beginning on day 11 with vehicle, roflumilast at 5.0 mg/kg once daily, pirfenidone at 100.0 mg/kg three times daily, or nintedanib 100.0 mg/kg two times daily. Mice were weighed daily (A) and on day 21, lungs were harvested and analyzed for total collagen content using an assay for hydroxyproline (B). Data are reported as mean ± SEM (*n* = 6–10 per group). Weight curves are analyzed with a two‐way ANOVA + Tukey multiple comparison tests (^#^ *P* ≤ 0.05 for DTR+:DT roflumilast and pirfenidone‐treatment versus DTR+:DT nintedanib treatment, and hydroxyproline is analyzed with a one‐way ANOVA + Tukey multiple comparison tests.

After establishing a therapeutic benefit of roflumilast and Compound 1 in reducing type II AEC‐injury‐induced fibrosis, we next compared the efficacy of PDE4 inhibition to treatment with the two FDA‐approved drugs for IPF (pirfenidone and nintedanib). DT was administered for 14 days according to our standard protocol. Beginning on day 11, subsets of DT‐injured DTR+ mice were treated with vehicle, roflumilast (5 mg/kg once daily), pirfenidone (100 mg/kg 3 times daily), or nindetinib (100 mg/kg twice daily). For pirfenidone and nintedanib, doses were chosen based on PK profiles and published data in the bleomycin model. The mice were weighed daily, and on day 21, lungs were harvested for lung collagen content. Consistent with our prior results, we found that roflumilast blunted the weight loss induced by DT administration in the DTR+ animals. Pirfenidone also reduced weight loss compared to the vehicle‐treated group. In contrast, therapeutic administration of nintedanib accentuated weight loss in this fibrosis model (Fig. 10A, *P* ≤ 0.05 on day 20–21 for roflumilast‐ and pirfenidone‐treated mice compared to the nintedanib‐treated group). Measurements of lung hydroxyproline revealed that all three drugs effectively reduced type II AEC‐injury‐induced fibrosis, and there was no difference in the antifibrotic efficacy of these agents at the given doses (Fig. 10B).

## Discussion

Diseases that are characterized by tissue fibrosis constitute major clinical challenges. Therefore, the approval of the first two drugs for patients with IPF, the prototypic scarring disorder of the lung, created excitement in the pulmonary community (King et al. 2014; Richeldi et al. 2014). Despite this recent advance, there is still a desperate need for additional well‐tolerated and efficacious treatments, and exploring the antifibrotic effect of already approved drugs with acceptable safety profiles offers an efficient approach to meet this demand. In this regard, we investigated the antifibrotic activity of both prophylactic and therapeutic PDE4 inhibitor administration in a type II AEC injury model of lung fibrosis. We found that three different members of this drug class including roflumilast (which is FDA approved for the treatment of COPD) and the novel agent The efficacy of Compound 1 in reducing lung fibrosis and blunting the associated weight loss was similar to roflumilast and piclamilast. Although roflumilast is known to show a body weight lowering effect in the clinical setting, treatment with the tested PDE4 inhibitors has a beneficial effect on weight loss in our fibrosis model compared to nintedanib and, less so, pirfenidone. Importantly, the antifibrotic activity of PDE4 inhibition in this model was equivalent to these two FDA‐approved therapies for IPF.

A large number of potential antifibrotic therapies have been shown to reduce fibrosis following bleomycin‐induced lung injury (Moeller et al. 2008). Based on their promising effect in this rodent model, a subset of these agents has been tested in well‐designed clinical trials with frustrating results. This lack of translation has led the fibrosis research community to question the utility of the bleomycin model in predicting the efficacy of prospective drugs. It should be noted, however, that the antifibrotic benefit of the majority of these drugs in the bleomycin model was established with preventative/prophylactic dosing. Because bleomycin induces inflammation which is a key element of the lung injury in this model, prophylactic administration of any drug that alters this inflammatory response is likely to impact fibrosis even if the agent lacks specific antifibrotic activity.

In light of this limitation of bleomycin, we chose to employ our type II AEC‐targeted injury model of lung fibrosis to evaluate the efficacy of the PDE4 inhibitors. This model shares pathogenic features with the human disease. For example, histologic studies consistently demonstrate type II cell defects including hyperplasia and apoptosis in patients with IPF (Kawanami et al. 1982; Katzenstein 1985; Kasper and Haroske 1996). Also, AEC‐specific gene mutations including surfactant proteins A2 and C are linked to familial lung fibrosis (Thomas et al. 2002; Wang et al. 2009). The limited and specific inflammatory response that occurs in this model may also better mimic the human disease (Osterholzer et al. 2013). To further enhance the translatability of our findings, we tested the efficacy of the PDE4 inhibitors not only in a preventative treatment regimen but also with therapeutic dosing (i.e., day 11–21). Although the potent effect of the PDE4 inhibitors in mitigating type II AEC injury‐induced fibrosis should motivate the further development of this class of drugs for patients with IPF, we acknowledge that the predictive value of our model of fibrosis requires validation. It should also be mentioned that the two approved therapies for IPF (nintedanib and pirfenidone) comprise a small group of drugs that, when administered therapeutically, reduced bleomycin‐induced fibrosis, suggesting that results from the bleomycin model, in the right context, can also guide clinical research decisions (Kakugawa et al. 2004; Chaudhary et al. 2007). Furthermore, nintedanib and pirfenidone significantly reduce fibrosis in both the bleomycin and type II AEC injury models, supporting the notion that efficacy in several distinct models of fibrosis helps predict translatability to human disease.

Multiple antifibrotic mediators including PGE_2_, prostacyclin, and adenosine signal through cAMP, motivating our interest in evaluating the efficacy of PDE4 inhibitors in our animal model of fibrosis. Among the pleiotropic antifibrotic effects of PGE_2_, it has been shown to protect the lung epithelium. For example, prior studies reveal that PGE_2_ inhibits Fas ligand‐induced apoptosis of type II AECs isolated from fibrotic lungs (Maher et al. 2010). This lipid mediator also promotes *in vitro* wound repair of airway epithelial cells (Savla et al. 2001). In our studies, treatment with Compound 1 effectively increased cAMP levels in an alveolar epithelial cell line, and we observed that the *in vivo* administration of this drug significantly reduced the plasma levels of SP‐D following targeted type II AEC injury in mice. Of note, SP‐D is increased in the serum of patients with IPF, and several studies indicate that this molecule, either alone or in conjunction with other proteins, functions as a diagnostic and prognostic biomarker in IPF (Takahashi et al. 2000; Greene et al. 2002; White et al. 2016). Although the mechanism by which serum levels of SP‐D are increased in IPF and other lung fibrotic disorders remains unknown, ongoing type II AEC injury in conjunction with hyperplasia likely contributes (Borensztajn et al. 2013; Nishikiori et al. 2014). The PDE4 inhibitor‐mediated decrease in the plasma concentration of SP‐D after the cessation of DT supports an effect of this class of drugs on the alveolar epithelium as an element of their antifibrotic activity.

In addition to promoting the integrity of the alveolar epithelium, PGE_2_ as well as prostacyclin analogs have been found to hinder a myriad of fibroblast functions. Specifically, PGE_2_ and treprostinil inhibit TGF‐*β*‐induced myofibroblast differentiation and promote the reversal of differentiated myofibroblasts to fibroblasts (Garrison et al. 2013; Epa et al. 2015; Corboz et al. 2018). In addition, PGE_2_ impedes the proliferation and migration of fibroblasts and also increases their susceptibility to apoptosis (Huang et al. 2009; Maher et al. 2010). In the setting of lung fibrosis, these antifibrotic activities of PGE_2_ are mitigated by a downregulation of its receptor in addition to an inhibition of its synthesis (Huang et al. 2010; Gabasa et al. 2013). Through the preservation of intracellular cAMP levels, PDE4 inhibitors have the capacity to augment the residual inhibitory actions of PGE_2_ on fibroblasts, and indeed, prior studies have found that roflumilast slows migration and reduces three‐dimentional gel contraction by these cells (Togo et al. 2009). In this study, we extend these findings by demonstrating that Compound 1‐mediated PDE4 inhibition decreases *in vitro* fibroblast expression of several profibrotic mediators including Col1a1, Fn, and PAI‐1. Of note, we did not find a significant PDE4 inhibitor‐induced reduction in the expression of these same genes *in vivo* although we did observe a trend for decreased expression of Col1a1, Fn. This lack of a statistically significant effect on profibrotic gene expression may be a reflection of the chosen time point for these studies, as we have previously shown that lung collagen content does not appreciably increase beyond day 21 in the type II AEC injury model (Sisson et al. 2010). Our findings might also be attributed to the dilution of a PDE4 inhibitor‐mediated effect in a specific cell population (i.e., fibroblast) resulting from the assessment of the whole lung RNA. We more likely would have detected a significant effect of PDE4 inhibition on profibrotic gene expression if measured specifically in fibroblasts on day 14 when collagen production is ongoing.

Beyond its effect of the alveolar epithelium and fibroblasts, it is also plausible that PDE4 inhibition limited fibrosis following type II AEC injury through an anti‐inflammatory effect. Notably, our prior studies have implicated a specific monocyte/macrophage predominant inflammation in the development of type II AEC‐injury‐induced fibrosis. In this study, we found that Compound 1 treatment significantly reduced the plasma levels of a subset of chemokines that are upregulated in pulmonary fibrosis and/or are influenced by cAMP signaling. Specifically, CXCL5/LIX concentrations were found to be increased in the lavage fluid of systemic sclerosis patients with associated interstitial lung disease (Hesselstrand et al. 2013), and elevated levels of CCL5/RANTES were measured in the BALF of IPF patients (Kodama et al. 1998). Furthermore, roflumilast treatment of lung fibroblasts has been found to decrease CCL5/RANTES and CXCL10/IP‐10 expression (Tannheimer et al. 2012). Finally, forskolin‐mediated induction of cAMP levels was shown to inhibit CCL11/eotaxin production by bronchial epithelial cells (Chu et al. 2010). In addition to its effect on plasma chemokine levels, we also found that PDE4 inhibition decreased the day 21 lung expression levels of TNF*α*. Prior studies using adenoviral‐mediated gene transfer suggest a direct role for this cytokine in fibrogenesis (Sime et al. 1998). On the other hand, the expression of TNF*α* in the alveolar compartment in the setting of a second hit (e.g., bleomycin) actually limited the fibrotic response (Fujita et al. 2003). Furthermore, the intratracheal instillation of recombinant TNF*α* to bleomycin‐injured mice with established fibrosis resulted in improvement in the severity of scarring (Redente et al. 2014). These disparate results suggest that the effect of TNF*α* on fibrosis is complex and may depend on the timing and location of its expression. Ultimately, future studies are necessary to establish a causal link between the PDE4 inhibition‐mediated reduction in cytokine/chemokine levels and the amelioration of fibrosis. However, the efficacy of both Compound 1 and roflumilast when initiated at a time point when the monocyte/macrophages have already maximally accrued suggests that the antifibrotic effect of PDE4 inhibition occurs predominantly through mechanisms other than blocking inflammation.

In conclusion, we have found that PDE4 inhibition significantly reduces lung fibrosis induced by targeted type II AEC injury. The antifibrotic activity of this class of drugs appears to be mediated through an array of cellular effects that promote the integrity of the alveolar epithelium and inhibit profibrotic gene expression by lung fibroblasts. The equal potency of therapeutic PDE4 inhibition compared to the FDA‐approved drugs in a model of lung fibrosis that shares pathogenic features with the human disease serves as strong motivation to investigate the efficacy of roflumilast or Compound 1 in patients with IPF. The absence of CYP inhibitory activity of Compound 1 suggests that it will have fewer drug–drug interactions than roflumilast and may be a more attractive candidate for early phase clinical trials.

## Conflict of Interest

None declared.

## Data Accessibility
